# Diagnostic accuracy, feasibility and acceptability of stool-based testing for childhood tuberculosis

**DOI:** 10.1183/23120541.00710-2023

**Published:** 2024-05-20

**Authors:** Bazezew Yenew, Petra de Haas, Yohannes Babo, Getu Diriba, Bihil Sherefdin, Ahmed Bedru, Ben Tegegn, Tilaye Gudina, Tadesse Getahun, Saro Abdella, Degu Jerene, Eveline Klinkenberg, Edine Tiemersma, the ASTTIE study group

**Affiliations:** 1 Ethiopian Public Health Institute, Addis Ababa, Ethiopia; 2 KNCV Tuberculosis Foundation, The Hague, The Netherlands; 3 KNCV Tuberculosis Foundation, Addis Ababa, Ethiopia; 4 Addis Ababa City Health Bureau, Addis Ababa, Ethiopia; 5 National Tuberculosis and Leprosy Control Program of Ethiopia, Addis Ababa, Ethiopia; 6 Yekatit 12 Hospital Medical College, Addis Ababa, Ethiopia; 7 Department of Global Health and Amsterdam Institute for Global Health and Development, Amsterdam University Medical Centers, Amsterdam, The Netherlands; 8 These authors contributed equally; 9 For a list of the ASTTIE study group members see the Acknowledgements

## Abstract

**Background:**

Childhood tuberculosis (TB) diagnosis remains challenging, partly because children cannot provide sputum. This study evaluated the diagnostic accuracy of the Simple One-Step (SOS) stool method with Xpert MTB/RIF Ultra (Xpert-Ultra) for childhood TB compared to culture and Xpert-Ultra on a respiratory sample (RS) and clinical diagnosis. It also assessed the feasibility and acceptability of stool testing according to laboratory staff, and caregivers’ sample preference.

**Methods:**

We enrolled children (≤10 years) with presumptive pulmonary tuberculosis in Ethiopia. RS was tested using Xpert-Ultra and culture; stool samples were tested using the SOS stool method with Xpert-Ultra. Laboratory staff and caregivers’ opinions were assessed using standardised questionnaires.

**Results:**

Of the 898 children enrolled, 792, 832 and 794 were included for assessing the diagnostic accuracy of SOS stool with Xpert-Ultra against culture, RS Xpert-Ultra and clinical diagnosis, respectively, yielding sensitivity estimates for SOS stool with Xpert-Ultra of 69.1% (95% confidence interval (CI) 56.0–79.7%), 76.8% (95% CI 64.2–85.9%) and 59.0% (95% CI 47.9–69.2%), respectively. The specificity was ≥98.8% for all comparisons. The rate of non-determinate test results was 2.8% after one repeat test. According to laboratory staff, stool collection was feasible and acceptable and the SOS stool method was easy to perform. Most caregivers (75%) preferred stool for TB diagnosis over RS.

**Conclusion:**

This study shows that SOS stool Xpert-Ultra testing offers a good alternative to RS testing for TB in children who cannot spontaneously produce a sputum sample and would otherwise need to undergo invasive procedures to obtain RS for diagnosis.

## Shareable abstract

**Simple One-Step (SOS) stool Xpert-Ultra testing is a good alternative to respiratory sample testing to diagnose tuberculosis in children, especially for children who cannot spontaneously produce a sputum sample** https://bit.ly/3T7uvpE

## Introduction

Tuberculosis (TB) continues to result in significant morbidity and mortality in children in low- and middle-income countries, partly due to challenges in diagnosis [1]. To detect TB in children, highly sensitive diagnostic methods that use noninvasive specimens are preferred [2]. Since 2020, the World Health Organization (WHO) recommends stool for diagnosing TB in children using the Xpert MTB/RIF (Xpert) [2] and Xpert MTB/RIF Ultra (Xpert-Ultra) assays [3].

The Simple One-Step (SOS) stool method [4] is one of the recommended stool processing methods [5]. This method does not require any additional supplies or equipment other than those needed for respiratory specimen Xpert testing, is easy to use, cost-effective to implement [3, 6, 7] and not sensitive to minor deviations from the protocol [8]. Head-to-head comparison studies, some involving stool spiked with *Mycobacteria*, in which the SOS stool method was compared against other stool processing methods, concluded that the sensitivity and specificity of all methods were similar [3, 6, 9, 10].

Pilot studies in Ethiopia and Vietnam have shown that the SOS stool method can be successfully implemented for routine diagnosis of TB in children and adults living with HIV [11–13]. Stool Xpert-Ultra testing is expected to increase access to bacteriologically confirmed TB diagnosis in children and reduce mortality [7], but evidence on its diagnostic performance remains limited. Thus, to demonstrate the diagnostic accuracy of the SOS stool method for diagnosing TB in children under routine settings, we evaluated the SOS stool method for Xpert-Ultra against Xpert-Ultra and culture on a respiratory sample (RS) from the same child. We also assessed laboratory staff's perspectives on the feasibility and acceptability of stool-based testing for TB diagnosis and children's caregivers’ sample preferences.

## Materials and methods

### Study setting and population

A prospective study was conducted in Addis Ababa and Oromia region, Ethiopia, from December 2018 to August 2021 in 38 healthcare facilities (HCFs): 18 primary health centres and 20 hospitals (four primary, eight secondary and eight tertiary levels). The sites were selected based on high TB notification rate, expertise in childhood TB diagnosis and onsite availability of the GeneXpert instrument. Consecutive children aged 10 years or below with signs and/or symptoms of pulmonary TB, including cough ≥2 weeks (or less for children with HIV infection), fever ≥2 weeks, night sweats, unexplained weight loss or failure to gain weight and history of contact with a TB patient [14], were eligible for enrolment.

### Training

At each study site, a medical director and two clinicians received 2 days of sensitisation training. Two laboratory staff per HCF received 4 days of training including practical training on the SOS stool processing method. Training was conducted by clinicians and senior laboratory experts involved in the development and validation of the SOS stool method. Standardised operating procedures and job aids were provided for each site.

### Specimen collection

For each child, at least one RS (either spontaneously expectorated sputum or nasogastric aspirate (NGA)) and one stool specimen were requested. NGA samples were collected the following day by trained healthcare workers (HCWs, *i.e.* clinicians/nurses) after overnight fasting per standard of care. If the sample volume was <3 mL, it was transported to the Ethiopian Public Health Institute (EPHI) for combined Xpert-Ultra and culture testing. RS with larger volumes were split into two aliquots after or during collection, one of which was tested with Xpert-Ultra at the site and the other transported to EPHI for culture testing.

Stool specimens were collected at the HCF by a caregiver or nurse. If defecation onsite was not possible, the caregiver was asked to collect a stool specimen at home and store it in a cool and dark place until return to the laboratory within 24 h of collection. Ideally, RS and stool samples were stored refrigerated (2–8°C) at the HCF until testing or transport to EPHI within 3 days of collection, using the routine cold chain transport system.

### Sample size

Assuming a sensitivity of 60% for Xpert-Ultra testing on stool using the SOS stool method compared to Mycobacteria Growth Indicator Tube (MGIT) culture on RS [10, 15, 16], a marginal error of 10% and a study power of 80%, and using the approach of Hess *et al.* [17], we calculated that 95 children with *Mycobacterium tuberculosis* (MTB) would need to be included. This corresponds to recruiting 1500 children with presumptive TB if 6.5% of these would be diagnosed with culture-confirmed TB [10].

### Laboratory procedures

#### Respiratory sample

Onsite RS Xpert-Ultra testing was done following the standard protocol for sputum Xpert testing. At EPHI, the sample was decontaminated using the NALC/NaOH method, and the sediment was inoculated into MGIT and Löwenstein–Jensen (LJ) cultures [18]. RS Xpert-Ultra test was conducted on the leftover sediment (GeneXpert Dx System Operator Manual, Cepheid, Sunnyvale, CA, USA). If the Xpert-Ultra result was non-determinate (*i.e.* invalid, error or no result), the test was repeated once using the leftover sample reagent–sample mixture. If a repeat test was not possible or if a repeat test was non-determinate, the non-determinate result was accepted. Any result of MTB detected, including a trace call, was regarded as MTB-positive, per WHO recommendations [2].

For RS culture testing, if no growth was observed within 42 (MGIT) or 56 days (LJ) of incubation, the result was recorded as negative. If growth was observed, the bacteria were tested for acid-fast bacilli (AFB) and identified using SD Bioline TB Ag MPT64 test (Standard Diagnostics Inc., Kyonggi, South Korea). If AFB positive and MPT64 positive, the result was recorded as MTB; if AFB positive and MPT64 negative, it was recorded as non-tuberculous mycobacteria (NTM); and if AFB negative and blood agar showed growth, the test was considered contaminated [18].

#### Stool sample

Stool samples were processed using the SOS stool method [10]. Briefly, around 0.8 to 1.0 g of formed/semi-formed or 2 mL of stool taking the shape of the container was added to the sample reagent bottle (Cepheid), which was then vigorously shaken for 30 s, incubated for 10 min, shaken again for 30 s and incubated for another 10 min. Then, 2 mL of the upper layer of supernatant was transferred to the Xpert-Ultra cartridge for testing. In the case of non-determinate Xpert-Ultra results, the test was repeated once (if possible) using a leftover sample. Any result of MTB detected, including trace call, was regarded as MTB-positive.

### Clinical diagnosis

Clinicians diagnosed TB in children according to the prevailing national guideline [14], based on a combination of the outcomes of bacteriological tests on RS, clinical features of TB, contact history and supportive evidence from investigations such as chest radiograph (CXR), if requested. Tuberculin skin testing and interferon gamma release assays were not routinely available. Abnormalities suggestive of TB on CXR included hilar or mediastinal lymph node enlargement with opacification, miliary mottling, cavitation, and pleural or pericardial effusion. The Xpert-Ultra stool result was not considered, as at the time of the study, stool was not included in the national guideline for the diagnosis of TB in children in Ethiopia [14].

### Acceptability and feasibility

The caregivers accompanying children participating in the study were interviewed using a standardised questionnaire with questions about the acceptability and the respondent's sample preference for TB diagnosis. Self-administered questionnaires were distributed to laboratory staff who conducted stool testing. The questions covered the acceptability of stool as a sample and the feasibility of the SOS stool method.

### Data collection

Data was collected from children using standard forms capturing demographic information, and disease- and sample-specific data, detailed test results, diagnostic decisions and treatment details.

For the acceptability and feasibility of the SOS stool method, data were collected from caregivers on their age, relationship with the child, experience with stool sample collection and sample preference for TB diagnosis. The information collected from laboratory staff included work-related experience, willingness to work with stool and opinion about the SOS stool method by asking about their level of agreement with statements about each step of the method on a 5-point scale, ranging from fully agree to fully disagree.

### Data management and statistical analysis

Data were entered at EPHI into preformatted EpiData data sheets containing automated (cross) checks (EpiData data version 3.1; www.epidata.com). Missing and inconsistent data were resolved with the sites. All data were further validated in Stata/SE 15.1 for Windows (www.stata.com) (StataCorp LLC, College Station, TX, USA). Data were analysed in Stata by applying statistical tests where appropriate, including chi-square and Fisher's exact test, proportion test for equality of proportions, Cochran–Armitage test for trend and Spearman's rank correlation test. We calculated Wilson confidence intervals around the diagnostic accuracy estimates. A p-value <0.05 was regarded statistically significant. We estimated the diagnostic accuracy (sensitivity, specificity and concordance rate) of Xpert-Ultra stool testing against Xpert-Ultra RS testing, culture (MGIT, or LJ result if MGIT was contaminated or did not detect MTB growth whereas LJ did), clinical diagnosis and any bacteriological confirmation on the RS (*i.e.* MTB detected in any of MGIT, LJ or Xpert-Ultra). For each comparison, we included records with valid results for both index and reference tests and calculated 95% Wilson's confidence intervals around the diagnostic accuracy estimates [19].

### Ethical considerations

This study received ethical approval from the EPHI Institutional Review Board (EPHI-IRB-134–2018). Caregivers provided written informed consent for their child's participation in the study, and separate informed consent to participate in the feasibility and acceptability interview. The laboratory staff also provided written informed consent prior to completing the questionnaire. No personal identifiers were included in electronic datasets. A unique participant identification code was used to merge data across forms. Personal identifiers were used only to link laboratory results to children for care and management when needed.

## Results

### Characteristics of population

**FIGURE 1:**
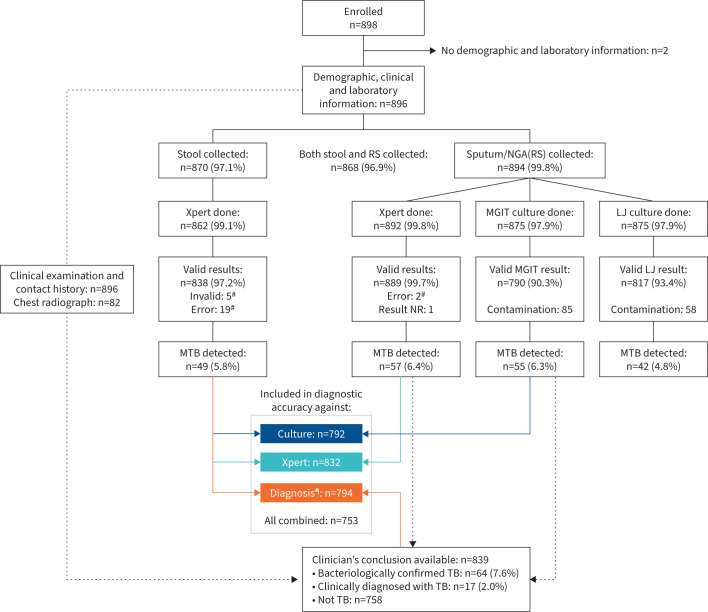
Study flow chart for the diagnostic accuracy study. LJ: Löwenstein–Jensen; MGIT: Mycobacterium Growth Indicator Tube; MTB: *Mycobacterium tuberculosis*; NGA: nasogastric aspirate; NR: not recorded; RS: respiratory sample (*i.e.* either spontaneously expectorated sputum or NGA); TB: tuberculosis; Xpert: Xpert MTB/Rif Ultra. ^#^: this is the result after a maximum of one repeat. The test was repeated if the initial result was non-determinate, *i.e.* error, invalid, or no result, and enough material was available for repeating the test. ^¶^: the diagnostic accuracy of Xpert stool testing was compared against the clinician's diagnosis, defined as TB (yes/no). Clinical diagnosis included both bacteriologically confirmed TB and TB diagnosed based on clinical grounds only (no bacteriological confirmation). Note that the stool test result was not considered for the diagnosis of TB in this study.

Of 898 eligible children, 896 had complete clinical and laboratory forms (figure 1). The median age of the children was 2 years (25th–75th percentile, 1–5 years) and 53.0% were male; for 85.1%, cough for >2 weeks was reported. A total of 82 (9.2%) children had a CXR result, of whom 50 (61.0%) had abnormalities suggestive of TB (table 1). Of 896 children, 894 (99.8%) provided an RS, and 870 (97.1%) provided stool, with 868 (96.9%) having both stool and RS collected (figure 1). Most (84%) RS were NGA; 93 (10.4%) samples had an estimated volume of <3 mL. Stool samples were mostly unformed (54.1%, table 1).

**TABLE 1:** General characteristics of the children included in the diagnostic accuracy study (n=896)

**Age, years** ^#^	2 (1–5)
**Age range, years**	
0 to <2	386 (43.1)
2 to <5	257 (28.7)
5 to 10	252 (28.2)
**Male** ^#^	475 (53.0)
**HIV status known**	476 (53.1)
Positive	63 (13.2)
Negative	413 (86.8)
**Other immunosuppressive condition** ^¶^	94 (10.5)
Of which (severe acute) malnutrition	54 (57.5)
**TB-suggestive signs and symptoms reported**	
Cough >2 weeks	760 (85.1)
Weight loss/failure to gain weight	618 (69.4)
Reduced playfulness/general malaise	505 (57.1)
Unexplained fever in past month	630 (70.6)
Night sweats	423 (59.2)
Enlarged lymph nodes in neck	139 (16.0)
Close contact with documented TB patient	368 (42.1)
Abnormal chest radiograph suggesting TB^+^	50 (61.0)
**Respiratory sample collected**	894 (99.8)
Spontaneous sputum	143 (16.0)
** **Volume mL	4.0±0.8
Nasogastric aspirate	751 (84.0)
** **Volume mL	4.0±1.0
**Stool sample collected**	870 (97.1)
**Appearance of stool sample collected**	
Formed	378 (43.5)
Unformed	471 (54.1)
Taking shape of container	12 (1.4)
Not specified	9 (1.0)

Data are presented as median (25th–75th percentile), n (%) and mean±sd. TB: tuberculosis. ^#^: age and sex missing for one participant. ^¶^: the immunosuppressive condition was not specified for all children. ^+^: a chest radiograph result was only available for 82 children. Where specified, the following were included more than once: gastrointestinal disease (8, 8.5%), pneumonia (6, 6.4%) and tonsilitis (4, 4.3%).

### Laboratory results

In total, 892 RS and 862 stool samples were tested using Xpert-Ultra. Valid Xpert-Ultra results were available for 889 out of 892 (99.7%) RS and 838 out of 862 (97.2%) stool specimens (p<0.001). MTB was detected in 57 out of 889 (6.4%) RS and 49 out of 838 (5.8%) stool samples (p=0.6) (figure 1). Rifampicin resistance was detected for one child both in the respiratory and stool sample (data not shown).

The initial Xpert-Ultra result was non-determinate for 11 out of 891 (1.2%) (three invalid, eight error) RS, and 50 out of 862 (5.8%) (12 invalid, 38 error) stool specimens (p<0.001). Most initial errors for stool were potentially related to processing, codes 2008 (n=25), 5006 (n=1) and 5007 (n=10). Errors 4016 and 5006, each occurring once, are unrelated to processing. The rate of initial non-determinate stool results dropped over time, from around 9% in the first 3 months to 2–3% in the last 12 months of the study (p_trend_=0.02; data not shown). The rate of non-determinate results after a maximum of one repeat is shown in table 2.

**TABLE 2:** Xpert-Ultra stool *versus* respiratory sample results obtained by Xpert-Ultra, MGIT and Löwenstein–Jensen (LJ) culture

	**MTB not detected**	**MTB detected**					**Invalid**	**Error**	**Not done**	**Total**
		**Trace**	**Very low**	**Low**	**Medium**	**High**				
**Overall (row %)**	789 (88.1)	12 (1.3)	9 (1.0)	19 (2.1)	7 (0.8)	2 (0.2)	5 (0.6)	19 (2.1)	34 (3.8)	896 (100)
**Sputum/NGA Xpert-Ultra result**										
** **MTB not detected	770 (97.6)	2 (16.7)	3 (33.3)	1 (5.3)	0 (0)	0 (0)	5 (100)	19 (100)	32 (94.1)	832 (92.9)
** **Trace	5 (0.6)	3 (25.0)	1 (11.1)	1 (5.3)	1 (14.3)	0 (0)	0 (0)	0 (0)	0 (0)	11 (1.2)
** **MTB detected, very low	6 (0.8)	2 (16.7)	0 (0)	3 (15.8)	0 (0)	0 (0)	0 (0)	0 (0)	1 (2.9)	12 (1.3)
** **MTB detected, low	2 (0.3)	4 (33.3)	5 (55.6)	11 (57.9)	0 (0)	0 (0)	0 (0)	0 (0)	0 (0)	22 (2.5)
** **MTB detected, medium	0 (0)	0 (0)	0 (0)	2 (10.5)	3 (42.9)	0 (0)	0 (0)	0 (0)	0 (0)	5 (0.6)
** **MTB detected, high	0 (0)	1 (8.3)	0 (0)	1 (5.3)	3 (42.9)	2 (100)	0 (0)	0 (0)	0 (0)	7 (0.8)
** **Invalid	0 (0)	0 (0)	0 (0)	0 (0)	0 (0)	0 (0)	0 (0)	0 (0)	0 (0)	0 (0)
** **Error	2 (0.3)	0 (0)	0 (0)	0 (0)	0 (0)	0 (0)	0 (0)	0 (0)	0 (0)	2 (0.2)
** **Not done	4 (0.5)	0 (0)	0 (0)	0 (0)	0 (0)	0 (0)	0 (0)	0 (0)	0 (0)	4 (0.5)
**MGIT culture result**										
** **Negative	674 (85.4)	4 (33.3)	3 (33.3)	3 (15.8)	0 (0)	0 (0)	4 (80.0)	16 (84.2)	29 (85.3)	733 (81.8)
** **MTB	17 (2.2)	8 (66.7)	6 (66.7)	14 (73.7)	7 (100)	2 (100)	0 (0)	0 (0)	1 (2.9)	55 (6.1)
** **NTM	2 (0.3)	0 (0)	0 (0)	0 (0)	0 (0)	0 (0)	0 (0)	0 (0)	0 (0)	2 (0.2)
** **Not done	19 (2.4)	0 (0)	0 (0)	1 (5.3)	0 (0)	0 (0)	0 (0)	0 (0)	1 (2.9)	21 (2.3)
**LJ culture result**										
** **Negative	703 (89.1)	7 (58.3)	4 (44.4)	6 (31.6)	0 (0)	0 (0)	5 (100)	18 (94.7)	31 (91.2)	774 (86.4)
** **MTB	11 (1.4)	5 (41.7)	5 (55.6)	11 (57.9)	7 (100)	2 (100)	0 (0)	0 (0)	1 (2.9)	42 (4.7)
** **NTM	1 (0.1)	0 (0)	0 (0)	0 (0)	0 (0)	0 (0)	0 (0)	0 (0)	0 (0)	1 (0.1)
** **Not done	19 (2.4)	0 (0)	0 (0)	1 (5.3)	0 (0)	0 (0)	0 (0)	0 (0)	1 (2.9)	21 (2.3)

Data are presented as n (%); percentages are column percentages, unless otherwise indicated. MTB: *Mycobacterium tuberculosis*; NGA: nasogastric aspirate; NTM: non-tuberculous mycobacteria.

Out of 875 RS tested on culture, 55 and 42 showed MTB growth on MGIT and LJ, respectively, while 85 and 58 were contaminated (figure 1, table 2).

Table 2 compares the semi-quantitative Xpert-Ultra stool results with those of RS Xpert-Ultra and culture results. The semi-quantitative Xpert-Ultra result for stool tended to be lower than that of RS, although there was a strong correlation between them (Spearman's ρ=0.82, p<0.0001). A weaker correlation existed between stool Xpert-Ultra result and time to growth on culture (Spearman's ρ= −0.26, p<0.0001).

### Clinical diagnosis

Of 896 children, 57 (6.4%) had no information on clinical diagnosis. A total of 81/839 (9.7%) children were diagnosed with TB (figure 1). This was based on bacteriological confirmation for 64 (7.6%) children, while 17 (2.0%) had no MTB detected in their RS by any microbiological method and were diagnosed with TB on a clinical basis (supplementary table S1), one of whom had MTB detected in the stool sample only. Four children had MTB detected on culture but did not receive a documented clinical diagnosis, as culture results became available only weeks after the data collection forms were filled (supplementary table S2).

### Diagnostic accuracy

**FIGURE 2:**
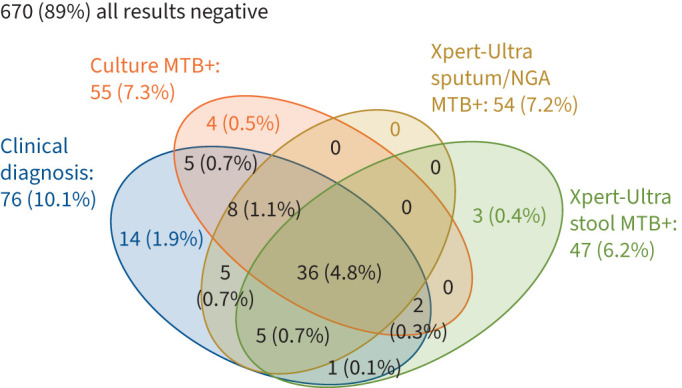
Venn diagram showing the concordance between the results of clinical diagnosis, culture Mycobacterium Growth Indicator Tube (MGIT), or Löwenstein–Jensen (LJ) if MGIT was contaminated, or LJ detected *Mycobacterium tuberculosis* (MTB) whereas MGIT did not, sputum/nasogastric aspirate (NGA) Xpert-Ultra and stool Xpert-Ultra, including all for whom data were available (n=753). MTB+: MTB-positive.

Figure 2 shows the concordance between RS Xpert-Ultra, culture and clinical diagnosis with the Xpert-Ultra stool results among 753 children with all results available. For six children, Xpert-Ultra detected MTB in stool but not RS; for four of these, MTB was not detected by culture (figure 2). All four children had a contact history of TB and/or CXR abnormalities suggestive of TB and/or multiple TB-suggestive symptoms; one child was HIV positive (table 3). Moreover, 13 children had MTB detected by Xpert-Ultra on RS but not on stool.

**TABLE 3:** Details of four children with *Mycobacterium tuberculosis* complex (MTB) detected only in their stool sample^#^

**Sex**	**Age, years**	**Cough >2 weeks**	**Weight loss/failure to gain weight**	**Reduced playfulness**	**Fever**	**Drenching night sweats**	**Lymph nodes in neck enlarged**	**TB contact history**	**CXR suggesting TB**	**HIV status**	**Immuno-suppressive conditions other than HIV**	**Other relevant disease**	**Xpert-Ultra stool result**	**Xpert-Ultra sputum/NGA result**	**MGIT culture result**	**LJ culture result**	**Clinical diagnosis of TB**
**F**	<1							X	X	Unknown			MTB detected, very low	MTB not detected	Neg	Neg	No
**M**	<1	X	X		X	X		X	X	Unknown			MTB detected, trace	MTB not detected	Neg	Neg	No
**M**	3	X	X	X				X	X	Unknown			MTB detected, low	MTB not detected	Neg	ND	No
**F** ^¶^	5	X	X	X	X				Unknown	Pos			MTB detected, trace	MTB not detected	Neg	Neg	Yes

TB: tuberculosis; CXR: chest radiograph; NGA: nasogastric aspirate; MGIT: Mycobacterium Growth Indicator Tube; LJ: Löwenstein–Jensen; F: female; M: male; Neg: negative; ND: not done; Pos: positive. ^#^: an “X” indicates that the symptom or sign of TB was reported as present by the caregiver; if the field is left blank, the symptom, sign or condition was not reported. ^¶^: clinically diagnosed with TB (therefore, also included in supplementary table S1). Anti-TB treatment was not started for this child for unknown reasons. The other three children were not diagnosed with TB.

**FIGURE 3:**
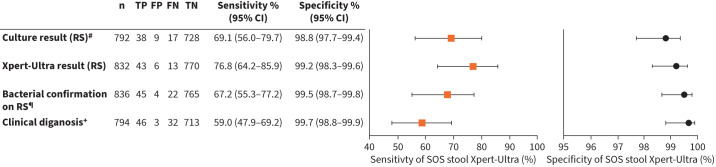
Diagnostic accuracy of Xpert-Ultra used on stool processed with the Simple One-Step (SOS) stool method as compared to the result of the respiratory sample on culture, and Xpert-Ultra, as well as clinical diagnosis. Squares and circles represent sensitivity and specificity point estimates, respectively; capped lines represent 95% Wilson confidence intervals. FN: false negative; FP: false positive; TN: true negative; TP: true positive. ^#^: the culture result was based on the Mycobacterium Growth Indicator Tube (MGIT) result. The Löwenstein–Jensen (LJ) result was taken if MGIT was contaminated, or if it did not detect *Mycobacterium tuberculosis* (MTB) whereas LJ did. ^¶^: bacteriological confirmation was defined as any detection of MTB in respiratory samples by culture or Xpert-Ultra. ^+^: clinical diagnosis included both bacteriologically confirmed tuberculosis (TB) and TB diagnosed based on clinical grounds only (no bacteriological confirmation). Note that the stool test result was not considered for the diagnosis of TB in this study.

Figure 3 shows the sensitivity and specificity estimates for Xpert-Ultra testing using the SOS stool method. The sensitivity of stool Xpert-Ultra was 69.1% (95% CI 56.0–79.7%) compared against culture (76.8%, 95% CI 64.2–85.9%) compared to the RS Xpert-Ultra result, and 59.0% (95% CI 47.9–69.2%) compared to clinical diagnosis. The specificity of stool Xpert-Ultra was ≥98.8% for all comparisons (figure 3). The concordance of the SOS Xpert-Ultra testing was 96.7% (95% CI 95.2–97.8%), 97.7% (95% CI 96.5–98.5%) and 95.6% (95% CI 93.9–96.8%) compared to culture, RS Xpert-Ultra and clinical diagnosis, respectively (data not shown). The diagnostic accuracy was not statistically significantly different for children aged below compared to those above 5 years (supplementary table S3). Excluding RS with a volume of <3 mL or unknown volume (n=94) from the analyses did not affect the statistical significance of estimates (data not shown).

### Feasibility and acceptability

In total, 569 (63.5%) caregivers filled the feasibility and acceptability questionnaire. Caregivers were more likely to fill the questionnaire if the child submitted sputum instead of NGA, submitted stool (*versus* no stool), was not HIV positive or had a clinical diagnosis form completed by the clinician-in-charge during the follow-up visit for diagnosis (*versus* no form) (supplementary table S4). Also, caregivers’ response rate varied by recruiting site (data not shown).

The questionnaire responses indicated that 75.0% of the caregivers preferred stool above sputum for TB diagnosis; however, 32.2% of them mentioned having experienced difficulties with collecting stool on-the-spot (data not shown).

**FIGURE 4:**
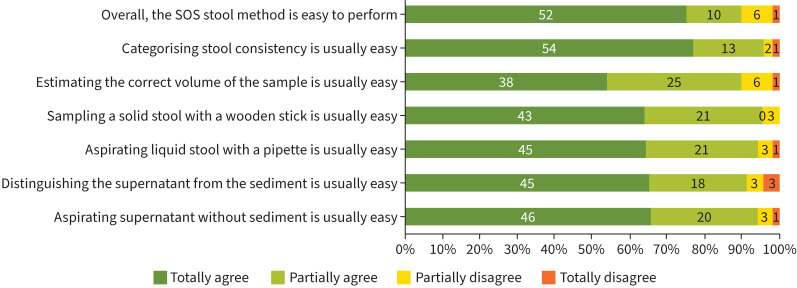
Feasibility of working with the Simple One-Step (SOS) stool method for Xpert-Ultra testing: opinions of 70 laboratory staff. The first and penultimate statements (from top to bottom in this figure) were answered by 69 out 70 participants, as one participant had no opinion on these.

Out of 76 trained laboratory staff, 70 (92.1%) completed the feasibility and acceptability questionnaire; 94.3% did not mind working with stool as a diagnostic sample (data not shown). Of 69 laboratory staff answering questions about the SOS stool method, 52 (75.4%) totally agreed, and 10 (14.5%) partially agreed that the method was easy to perform (figure 4). The most challenging steps were estimating the correct volume of stool, sampling from a solid stool with a wooden stick and distinguishing supernatant from sediment (figure 4).

## Discussion

The sensitivity of Xpert-Ultra for stool processed with the SOS stool method was 76.8% compared to the RS Xpert-Ultra result, but slightly lower when compared to culture (69.1%). The specificity of the test was ≥98.8% in all comparisons. The rate of non-determinate stool Xpert-Ultra results was acceptably low after one repeat test (2.8%). As a diagnostic sample, stool was preferred over RS by 75% of the caregivers and was well accepted as a diagnostic sample for TB by laboratory staff. Almost 90% of laboratory staff (partially) agreed that the SOS stool method is easy to perform.

The SOS stool method's sensitivity and specificity reported here comply with the optimal sensitivity of 66% and specificity of 98% for a rapid non-sputum-based test for detecting TB in children, as proposed by the WHO in their target product profile report [20]. The only other data published on diagnostic accuracy of SOS stool Xpert testing [21] yielded higher sensitivity (100%) and specificity (99%), but the confidence intervals were wide and overlapped with ours [11]. Using a reference standard (LJ culture) which is less sensitive than MGIT culture [22] may have contributed to the higher sensitivity estimate in that study [11]. We found a clear association between the bacillary load of the RS (determined by semi-quantitative Xpert-Ultra results), time to culture positivity and probability of MTB being detected in stool. The four children with MTB detected only in their stool but not in their RS (one of whom was clinically diagnosed with TB) had a bacillary load of low, very low or trace. All had multiple signs and/or symptoms of TB (table 3). This finding supports the current WHO recommendations that children with presumptive TB for whom MTB is detected in the stool should be treated for TB [3]. A study from Vietnam showed that young children would benefit from stool Xpert testing, and that combining stool with RS provided the highest diagnostic yield [13]. It is probably most cost-effective to first test stool from children with presumptive TB using Xpert-Ultra, and then test RS if TB is not detected in stool and TB is still highly presumed [7]. This could also reduce diagnostic delays, as stool can be easily collected at all levels of the healthcare system without distress [23] for the children, caregivers and HCWs.

The rate of non-determinate stool Xpert-Ultra test results dropped from 5.8% to 2.8% after one repeat and the error rate dropped from 4.4% to 2.1%. These values also dropped with time of study operation, but remained higher for stool than RS, which is likely due to the nature of stool in that it contains more PCR inhibitors and debris than sputum [8, 10]. Yet, the non-determinate and error rate remained below the maximum of 5%, respectively 3% for sputum, as recommended by the WHO for Xpert-Ultra testing [24]. Errors potentially related to stool processing (codes 2008, 5006 and 5007) were the most reported non-determinate results. In the pilot in Vietnam including 10 mostly high-level facilities, these error codes were also the most common and a similar error rate was observed [13]. However, in our study, 38 laboratories participated, of which 22 were primary level, >70 laboratory staff conducted the SOS stool method after receiving training from various trainers, and data collection was conducted over a longer period. This shows that the SOS stool method can be implemented in routine Xpert laboratories with minimal training. It also confirms our previous findings that this method is not very sensitive to minor deviations from the protocol [8]. Results from that study indicated that the initial error rate might drop if less stool is used for testing [8].

Stool was a well-accepted sample for diagnosis of TB among caregivers and laboratory staff. This is in line with data from Vietnam [13]. Sanogo *et al.* [23] reported that children tolerated stool collection much better than NGA. Despite this, almost one-third of parents reported issues with on-the-spot stool collection. Collection of rectal swabs from critically ill children who cannot defecate on-the-spot may be further explored to overcome this issue. While there are no data on rectal swabs, no difference was found in the sensitivity of Xpert applied on faecal swabs compared to stool samples [25, 26]. The SOS stool method was perceived as easy to perform by most laboratory staff, in line with data from Vietnam [13], and is seen as the most suitable method for global scale-up by experts from the WHO and Global Laboratory Initiative [5] and Jasumback *et al.* [6].

This study was conducted in urban and semi-urban areas in multiple HCFs, and SOS stool testing was integrated into routine diagnostic procedures for children. The precision of our diagnostic accuracy estimates was lower than anticipated because the desired sample size was not achieved due to the impact of the COVID-19 pandemic. Data on more children would be needed to get more precise estimates of sensitivity and specificity. However, based on previous work [11–13], we have no reason to assume that a larger sample size would have changed our overall conclusions. Our estimates of the diagnostic accuracy of SOS stool Xpert-Ultra testing against clinical diagnosis should be interpreted with caution as clinical diagnosis remains subjective. Per prevailing guidance [14], Xpert-Ultra stool results were not shared with the clinicians during study operations, and the diagnosis of TB was therefore missed in three children with MTB detected only in their stool (table 3). Unfortunately, we do not have follow-up data for these children and thus do not know if they were diagnosed and started TB treatment later. Since August 2021, stool is a recommended sample for the diagnosis of TB in children with presumptive pulmonary TB in Ethiopia [27].

The SOS stool Xpert-Ultra testing was accurate, feasible and acceptable to laboratory staff and caregivers. Given the fact that only 16% of the children in our population spontaneously expectorated a sputum sample, SOS stool Xpert-Ultra testing offers a good alternative to RS testing for TB in children who cannot spontaneously produce a sputum sample and, importantly, increases access to noninvasive testing for TB in children closer to their home. Many countries have started introducing stool-based testing by performing a pilot implementation, and some are in the scale-up phase. This will provide more insights into effective strategies to increase access to stool-based TB diagnostics.

## Supplementary material

10.1183/23120541.00710-2023.Supp1 **Please note:** supplementary material is not edited by the Editorial Office, and is uploaded as it has been supplied by the author.
Supplementary material 00710-2023.SUPPLEMENT
