# Supplementary material for: Diagnostic accuracy, feasibility and acceptability of stool-based testing for childhood tuberculosis
Source: ERJ Open Res. 2024 May 20;10(3):00710-2023. doi: 10.1183/23120541.00710-2023 (PMC11103712; doi:10.1183/23120541.00710-2023)
Supplement: Supplementary file 1 [file 00710-2023.SUPPLEMENT.pdf]

Supplementary Table 1. Details of 17 patients clinically diagnosed with TB.*

Sex	Age in years	Cough > 2 weeks	Weight loss	Reduced playfulness	Fever	Drenching night sweats	Lymph nodes in neck enlarged	TB contact history	CXR suggesting TB	HIV status	Immuno-suppressive conditions other than HIV	Other relevant disease	Xpert-Ultra stool result	Xpert-Ultra sputum/NGA result	MGIT culture result	LJ culture result
**F**	0.1	X	X	X	X			X	X	unknown		Pneumonia	ND	neg	neg	neg
**M**	0.3	X	X	X	X	X	X		X	unknown	SAM + LONS		neg	neg	neg	neg
**M**	1	X	X					X	X	neg			error	neg	neg	neg
**M**	1	X	X	X	X					neg	AGE + SAM	SCAP	neg	neg	neg	neg
**M**	1.3	X	X	X	X	X	X	X	X	neg			neg	neg	neg	neg
**F**	1.5	X	X	X	X	X			X	neg	SAM	CHF + asthma	neg	neg	neg	neg
**M**	1.8	X	X	X	X				X	neg			neg	neg	neg	neg
**M**	2	X	X	X	X	X	X	X	X	neg			neg	neg	neg	neg
**M**	3	X	X		X			X	X	neg	Marasmus		neg	neg	neg	neg
**F**	4	X	X	X	X	X	X	X	X	unknown			neg	neg	neg	neg
**F**	4	X	X	X	X	X	X		X	pos			neg	neg	neg	neg
**M**	5	X	X		X			X		neg			neg	neg	neg	neg
**F****	5	X	X	X	X				unknown	pos			trace	neg	neg	neg
**M**	7	X	X	X	X	X		X	X	neg			neg	neg	neg	neg
**F**	8		X	X				X		unknown			neg	neg	neg	neg
**M**	8	X	X	X	X	X			X	pos		SCAP	neg	neg	neg	neg
**M**	8	X	X	X	X			X	X	pos			neg	neg	neg	neg

* An 'X' indicates that the symptom or sign of TB was reported as present by the caregiver; if the field is left blank, the symptom, sign or condition was not reported. Abbreviations used: AGE, acute gastroenteritis; CHF, congestive heart failure; CXR, chest-X-ray; F, female; LJ, Löwenstein-Jensen; LONS: late-onset neonatal sepsis; M, male; MGIT: Mycobacterium growth indicator tube; ND, not done; NGA, nasogastric aspirate; neg, negative; pos, positive; SAM, severe acute malnutrition; SCAP, severe community-acquired pneumonia; TB, tuberculosis.
**Also included in Table 3 as *Mycobacterium tuberculosis* complex was detected in the stool of this child.

Supplementary Table 2. Results of participants with any type bacteriological confirmation of TB on the respiratory sample*'**.

Xpert-Ultra sputum/GA result	Xpert-Ultra stool result	MGIT culture result	LJ culture result	N	Remark
MTB not detected	MTB not detected	MTB+	MTB+	3	Recorded as TB not diagnosed on study form***
MTB not detected	MTB not detected	MTB+	not done	1	
MTB detected, trace	MTB not detected	neg	neg	2	
MTB detected, trace	MTB detected, >trace	neg	neg	1	
MTB detected, >trace	MTB not detected	neg	neg	3	
MTB detected, >trace	MTB detected, trace	neg	neg	2	
MTB detected, >trace	MTB detected, >trace	neg	neg	2	
MTB detected, >trace	MTB detected, >trace	neg	MTB+	1	
MTB not detected	MTB not detected	MTB+	neg	2	
MTB not detected	MTB detected, >trace	MTB+	neg	1	
MTB detected, trace	MTB not detected	MTB+	neg	1	
MTB detected, trace	MTB detected, >trace	MTB+	neg	1	
MTB detected, >trace	MTB not detected	MTB+	neg	2	
MTB detected, >trace	MTB detected, trace	MTB+	neg	3	
MTB detected, >trace	MTB detected, >trace	MTB+	neg	3	
MTB not detected	MTB not detected	MTB+	MTB+	3	
MTB not detected	MTB detected, >trace	MTB+	MTB+	1	
MTB detected, trace	MTB not detected	MTB+	MTB+	2	
MTB detected, trace	MTB detected, trace	MTB+	MTB+	3	
MTB detected, trace	MTB detected, >trace	MTB+	MTB+	1	
MTB detected, >trace	MTB not detected	MTB+	MTB+	3	
MTB detected, >trace	MTB detected, trace	MTB+	MTB+	2	
MTB detected, >trace	MTB detected, >trace	MTB+	MTB+	22	
MTB detected, >trace	not done	MTB+	MTB+	1	No stool sample available
MTB detected, >trace	MTB detected, >trace	Contaminated	Contaminated	1	
MTB detected, >trace	MTB detected, >trace	not done	not done	1	Sputum/NGA not sent to EPHI for culture
	**Total**			**68**	

*Abbreviations used: EPHI, Ethiopian Public Health Institute; LJ, Löwenstein-Jensen; MGIT: Mycobacterium growth indicator tube; MTB, *Mycobacterium tuberculosis* complex; MTB+, colonies on culture identified as *Mycobacterium tuberculosis* complex; N, number; neg: negative (no growth); NGA, nasogastric aspirate; TB, tuberculosis.

**Four additional children had MTB detected in stool, however, Xpert-Ultra stool result was not considered for a TB diagnosis, as stool Xpert (Ultra) testing was not recommended for the diagnosis of TB in children with presumptive TB.

*** At the time that the treating clinician made the diagnosis, culture results were not yet available. However, all clinicians treating a child with any *Mycobacterium tuberculosis* complex growth on culture were informed of the culture result and were encouraged to contact the child's caregivers and start the child on TB treatment. At least one of these children was traced and started on TB treatment.

Supplementary Table 3. Diagnostic accuracy of the SOS stool method with Xpert-Ultra compared to different reference standards, by age group*.

	Sensitivity, % (95% CI)			Specificity, % (95% CI)		
**Reference standard**	**<2 years**	**2-4 years**	**5-10 years**	**<2 years**	**2-4 years**	**5-10y**
**Culture**	53.3 (26.6 - 78.7)	77.3 (54.6 - 92.2)	72.2 (46.5 - 90.3)	98.4 (96.2 - 99.5)	99.5 (97.2 - 100)	98.6 (95.9 - 99.7)
**Sputum/NGA Xpert-Ultra**	66.7 (41.0 - 86.7)	85.0 (62.1 - 96.8)	77.8 (52.4 - 93.6)	99.4 (97.9 - 99.9)	99.1 (96.7 - 99.9)	99.1 (96.8 - 99.9)
**Clinical diagnosis**	52.2 (30.6 - 73.2)	64.3 (44.1 - 81.4)	59.3 (38.8 - 77.6)	99.4 (97.8 - 99.9)	99.5 (97.1 - 100)	100 (98.2 - 100)

*Abbreviations used: CI, confidence interval; NGA, nasogastric aspirate; SOS, simple one-step.

Supplementary Table 4. Factors associated with caregivers' willingness and ability to complete the self-administered questionnaire about experiences with collecting stool and sample preferences.

**Characteristic**	**Did not fill out the questionnaire (N=327)**		**Did fill out the questionnaire (N=569)**		**Total**	
	**N****	**%****	**N****	**%****	**N**	***p*-value*****
**Child's mean age (years) ± SD**	3.2	± 3.0	3.4	± 3.1		0.5
**Child's sex**						0.62
Male	170	52.0%	305	53.7%	475	
Female	157	48.0%	263	46.3%	420	
**Type of RS sample collected from child**						<0.001
Spontaneously expectorated sputum	31	9.5%	130	22.8%	161	
NGA	295	90.2%	438	77.0%	733	
Not collected	1	0.3%	1	0.2%	2	
**Stool collected from child?**						p<0.001
No	22	6.7%	4	0.7%	26	
Yes	305	93.3%	565	99.3%	870	
**TB diagnosed in child?**						0.44
No	247	91.5%	511	89.8%	758	
Yes	23	8.5%	58	10.2%	81	
**HIV status**						0.002
Positive	36	11.1%	27	4.8%	63	
Negative	140	43.1%	273	48.2%	413	
Unknown	149	45.8%	266	47.0%	415	
**Clinical diagnosis form available for the child?**						<0.001
No	28	8.6%	7	1.2%	35	
Yes	299	91.4%	562	98.8%	861	

* Abbreviations used: N, number; NGA, nasogastric aspirate; RS, respiratory sample; SD, standard deviation; TB, tuberculosis.

** Number, respectively percentage, unless otherwise indicated.

*** p-values were obtained using Chi-square testing if cell counts were 5 or above, and Fisher's exact test if lower than 5.
